# Lactoferrin Modulates Induction of Transcription Factor c-Fos in Neuronal Cultures

**DOI:** 10.3390/ijms24098373

**Published:** 2023-05-06

**Authors:** Marina Yu. Kopaeva, Asya M. Azieva, Anton B. Cherepov, Irina Yu. Zarayskaya

**Affiliations:** 1 National Research Center “Kurchatov Institute”, 1 Akademika Kurchatova Sq., 123182 Moscow, Russia; 2 Institute of General Pathology and Pathophysiology, 8 Baltiyskaya St., 125315 Moscow, Russia; 3 Research Institute of Normal Physiology Named after P.K. Anokhin, 8 Baltiyskaya St., 125315 Moscow, Russia

**Keywords:** human lactoferrin, transcription factor c-Fos, neuronal cultures, stimulation, cytoplasmic and nuclear localization

## Abstract

Lactoferrin (Lf) is a multifunctional protein from the transferrin family. Of particular interest is the ability of Lf to affect a wide range of neuronal processes by modulating the expression of genes involved in long-term neuroplasticity. The expression of the immediate early gene *c-fos* that is rapidly activated in response to external influences, and its product, transcription factor c-Fos, is widely used as a marker of long-term neuronal plasticity. The present study aims to examine the effect of human Lf on the induction of transcription factor c-Fos in the primary mouse neuronal cultures after stimulation and to determine the cellular localization of human Lf and its colocalization with induced c-Fos protein. Primary dissociated cultures of hippocampal cells were obtained from the brains of newborn C57BL/6 mice (P0-P1). On day 7 of culturing, human Lf was added to the medium. After 24 h (day 8 in culture), c-Fos protein was induced in cells by triple application of 50 mM KCl. c-Fos content was analyzed using the immunofluorescent method 2 h after stimulation. Stimulation promoted exogenous Lf translocation into the nuclei of cultured neuronal cells, which correlated with increased induction of transcription factor c-Fos and was accompanied by nuclear colocalization of these proteins. These results attest to the potential of Lf as a modulator of neuronal processes and open up new prospects in studying the mechanisms of the regulatory effects of lactoferrin on cell function.

## 1. Introduction

Lactoferrin (Lf) is a multifunctional protein from the transferrin family characterized by a high affinity for Fe^3+^. This globular glycoprotein is present in various mammalian secretory fluids, as well as in neutrophil granules [1,2]. Lf is actively studied in experimental models of human diseases as an antiallergic, immunomodulatory, and radioprotective agent [3,4,5], as well as a protein capable of attenuating the progression of neurodegenerative diseases and stimulating neuroregeneration [6,7,8,9].

Of particular interest is the ability of Lf to affect a wide range of neuronal processes by modulating the expression of genes involved in long-term neuroplasticity (BDNF, CREB, CAMK, etc.) [10,11]. It is now generally recognized that endogenous Lf in the brain is secreted only by activated microglia [7], and neurons could uptake it through the Lf-receptor-mediated pathway [12]. Some cells have receptors that allow them to bind Lf of other species [13]. It is known that endothelial capillary cells in mouse brain can internalize human Lf [14]. In our previous studies, highly specific binding sites for human lactoferrin (hLf) were revealed by immunohistochemical methods in the neuronal nuclei in mouse brain [15]. The expression of the immediate early gene *c-fos* that is rapidly activated in response to external influences, including stress, and its product, transcription factor c-Fos, is widely used as a marker of long-term neuronal plasticity [16,17,18]. The c-Fos protein is a genetic regulator for cellular mechanisms mediating the excitability and survival of neurons [19].

Transcription factors regulate the expression of specific target genes in response to signaling events on the cell membrane. *c-fos* encodes the transcription factor that regulates the activity of effector genes and the following long-term plasticity in neurons [18,20,21]. In cell cultures, the expression of immediate early genes can be induced by diverse stimuli. For instance, electrical stimulation significantly increases c-Fos expression in the neuronal culture [22] and in rat hippocampus [23], while KCl application was followed by the appearance of c-Fos protein in cultured hippocampal slices [24]. A possible relationship between Lf and c-Fos induction at the level of individual cells is still not explored.

Our aim was to study the effect of hLf on the induction of transcription factor c-Fos in primary mouse neuronal cultures after stimulation and to determine the cellular localization of hLf and possible colocalization of the exogenous protein with induced c-Fos protein.

## 2. Results

### 2.1. Dynamics of hLf Penetration into the Cytoplasm and Nucleus of Cells in the Mouse Primary Culture of the Hippocampus

**Figure 1:**
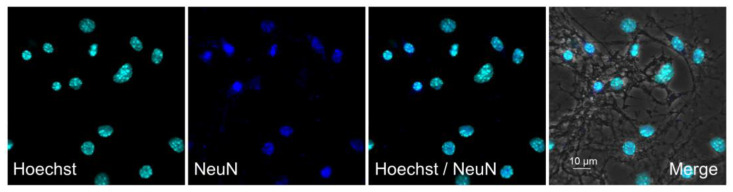
Immunofluorescent detection of NeuN, a marker of mature neurons (dark blue) in hippocampal cell cultures on day 7 of culturing. Primary dissociated cultures of hippocampal cells were obtained from the brains of newborn C57BL/6 mice (P0-P1). Nuclei were poststained with Hoechst (blue). Representative micrographs of cultures. Scale bar = 10 μm.

Primary dissociated cultures of hippocampal cells were obtained from the brain of newborn C57BL/6 mice (P0-P1). The Immunoreactivity of the cell culture to the neuronal marker NeuN indicated that ~85% of cells were neurons on day 7 of culturing (Figure 1), which is consistent with previous reports [25,26].

**Figure 2:**
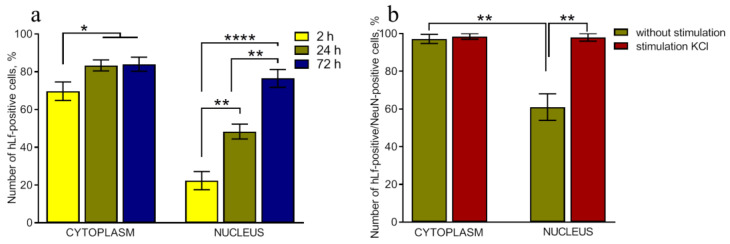
The dynamics of Lf penetration into the cytoplasm and nucleus of cells in the primary culture of the hippocampus. Primary dissociated cultures of hippocampal cells were obtained from the brains of newborn C57BL/6 mice (P0-P1). On day 8 of culturing, human Lf was added to the medium to make a final concentration of 1 mg/mL for 2, 24, or 72 h. Immunofluorescent staining was performed using monoclonal antibodies to human Lf. No hLf-positive cells were detected before Lf addition. (**a**). On day 8, culturing stimulation was carried out by adding 50 mM KCl (3 × 2 min with 5 min intervals) to the culture medium with complete medium replacement between the stimulations. Human Lf (to a final concentration of 1 mg/mL) was preliminary added for 24 h. Immunocytochemical analysis was performed 2 h after stimulation (**b**). *n* = 4–7 for each group. The data are presented as the mean ± SEM. * *p* < 0.05; ** *p* < 0.01; and **** *p* < 0.0001.

Immunofluorescent staining of non-stimulated cultures revealed both cytoplasmic and nuclear localization of hLf in cells 2, 24, and 72 h after it was added to the culture medium. Immunofluorescent staining was performed using monoclonal antibodies to hLf. No hLf-positive cells were detected before the addition of hLf. The protein quickly entered the cytoplasm and at 2, 24, and 72 h after addition to the culture it was detected in 70, 83, and 84% of cells (Figure 2a). Lf translocation into the nucleus took more time. The number of immunoreactive nuclei depended on the time of cell incubation with the protein: 2, 24, and 72 h after hLf addition to the cells, it was detected in 22, 48, and 72% of cells of intact culture (Figure 2a).

Moreover, 24 h after hLf addition to the culture, immunoreactivity was detected in the cytoplasm of 97% and nuclei of 61% of NeuN-positive cells. Stimulation with 50 mM KCl accelerated protein penetration into the nucleus and significantly increased this parameter to 98% (*p* < 0.01) after 2 h (Figure 2b).

### 2.2. Evaluation of Genomic DNA Fragmentation in Cultured Cells after Stimulation

In our work, damage to genomic DNA was assessed by the percentage of cells with DNA fragmentation from the total number of cells in culture [27]. We compared this parameter in groups KCl (2 and 24 h after stimulation) and PC (passive control).

**Figure 3:**
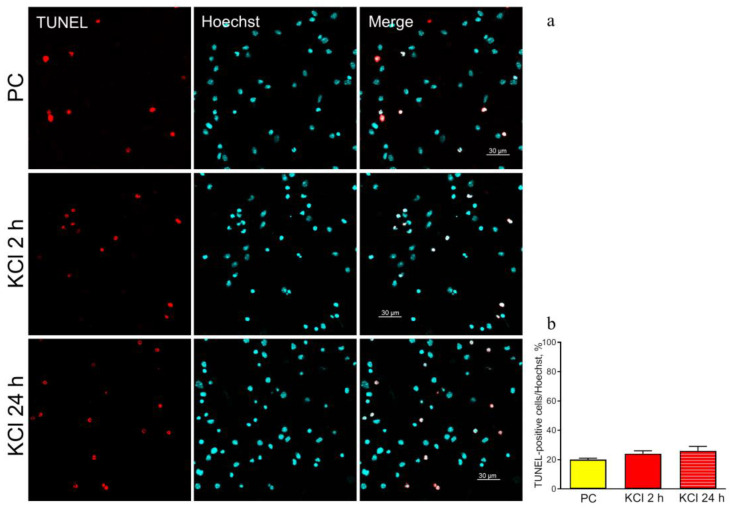
TUNEL staining (red) of hippocampal cell cultures 2 and 24 h after stimulation with 50 mM KCl. Representative micrographs of cultures in groups PC (passive control) and KCl. Nuclei were poststained with Hoechst (blue). Primary dissociated cultures of hippocampal cells were obtained from the brains of newborn C57BL/6 mice (P0-P1). On day 8 of culturing, stimulation was carried out by adding 50 mM KCl (3 × 2 min with 5 min intervals) to the culture medium with complete medium replacement between the stimulations. The PC groups were fixed simultaneously with other cultures without additional manipulations. Scale bars = 30 μm (**a**). Quantitative analysis of TUNEL-positive cells. *n* = 4 for each group The data are presented as the mean ± SEM (**b**).

Immunofluorescent analysis showed that on day 8 of culturing, the spontaneous level of TUNEL-positive cells in the PC group was 20%. Stimulation with 50 mM KCl did not significantly increase this parameter: 25 and 28% of TUNEL-positive cells 2 and 24 h after stimulation, respectively (Figure 3a,b).

### 2.3. Effect of hLf on the Expression of Transcription Factor c-Fos

**Figure 4:**
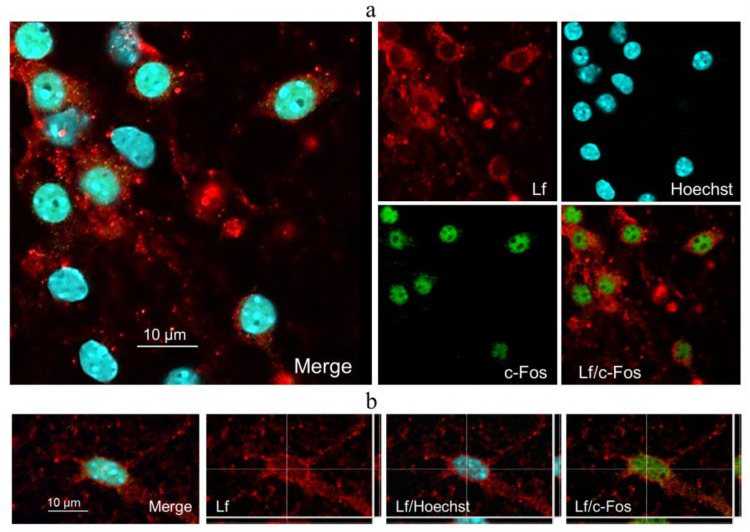
Immunofluorescent detection of c-Fos protein (green) and Lf (red) in hippocampal cell cultures on day 8 of culturing 2 h after stimulation. Nuclei were poststained with Hoechst (blue). Representative micrographs of cultures in group KCl+Lf. Primary dissociated cultures of hippocampal cells were obtained from the brains of newborn C57BL/6 mice (P0-P1). On day 8 of culturing, stimulation was carried out by adding 50 mM KCl (3 × 2 min with 5 min intervals) to the culture medium with complete medium replacement between the stimulations. Human Lf (to a final concentration of 1 mg/mL) was preliminary added for 24 h. Immunofluorescent staining was performed using monoclonal antibodies to hLf. Presence of hLf in the cytoplasm of all c-Fos-positive cells in the KCl+Lf group 2 h after stimulation (**a**). Colocalization of hLf inclusion and c-Fos protein was observed in some nuclei (**b**). Scale bars = 10 μm.

Immunofluorescent staining showed the presence of hLf in the cytoplasm of all c-Fos-positive cells in the KCl+Lf group 2 h after stimulation with 50 mM KCl (Figure 4a). The c-Fos protein had nuclear localization. In addition, colocalization of hLf inclusion and c-Fos protein was observed in some nuclei (Figure 4b).

**Figure 5:**
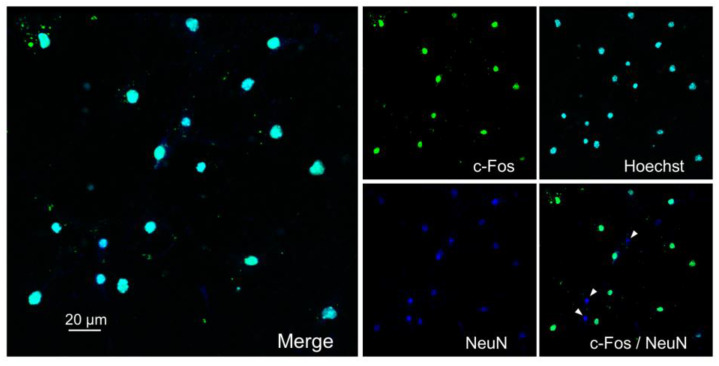
Immunofluorescent detection of c-Fos protein (green) and NeuN (dark blue) in hippocampal cell cultures on day 8 of culturing 2 h after stimulation. Nuclei were poststained with Hoechst (blue). Representative micrographs of cultures in group KCl+Lf. The arrows indicate NeuN-positive/c-Fos-negative cells. Primary dissociated cultures of hippocampal cells were obtained from the brains of newborn C57BL/6 mice (P0-P1). On day 8 of culturing, stimulation was carried out by adding 50 mM KCl (3 × 2 min with 5 min intervals) to the culture medium with complete medium replacement between the stimulations. Human Lf (to a final concentration of 1 mg/mL) was preliminary added for 24 h. Scale bar = 20 μm.

Two hours after stimulation, 69% of NeuN-positive cells were also c-Fos-positive (Figure 5).

**Figure 6:**
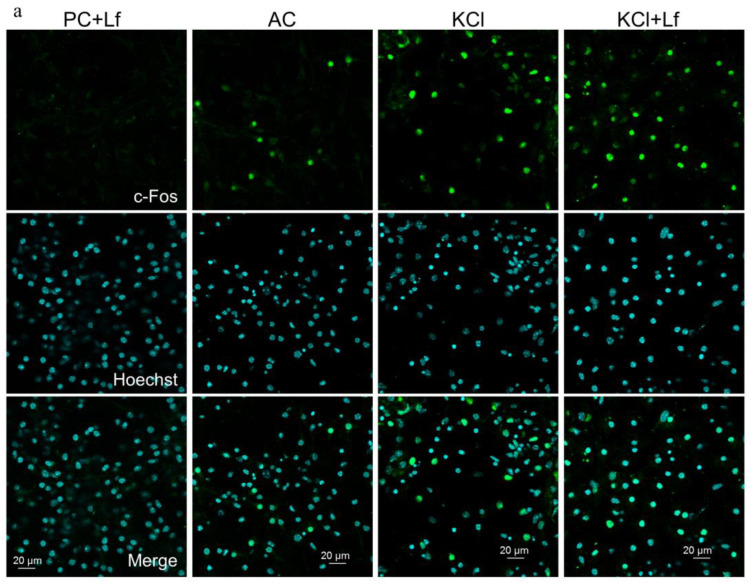

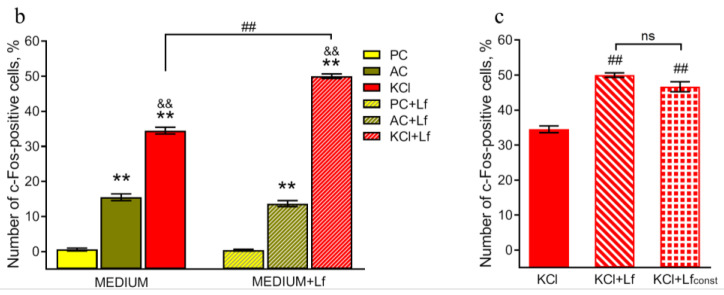
Immunofluorescent detection of c-Fos protein (green) in hippocampal cell cultures on day 8 of culturing 2 h after stimulation. Nuclei were poststained with Hoechst (blue). Representative micrographs of cultures in groups PC+Lf, AC, KCl, and KCl+Lf. Primary dissociated cultures of hippocampal cells were obtained from the brains of newborn C57BL/6 mice (P0-P1). On day 8, culturing stimulation was carried out by adding 50 mM KCl (3 × 2 min with 5 min intervals) to the culture medium with complete medium replacement between the stimulations. Human Lf was added to the culture medium of KCl+Lf, KCl+Lfconst, AC+Lf, and PC+Lf groups to a final concentration of 1 mg/mL 24 h before stimulation, medium changing, or fixation. In the KCl+Lfconst group, the concentration of hLf during medium changes between the stimulations was maintained constant until the fixation of the cultures. Cultures with 3-fold medium replacement were used as active controls. The passive control group was fixed simultaneously with other cultures without additional manipulations. Scale bars = 20 μm (**a**). Quantitative analysis of c-Fos protein induction in hippocampal cell cultures on day 8 of culturing 2 h after stimulation with 50 mM KCl. ** *p* < 0.0001—differences from the corresponding passive control groups; && *p* < 0.0001—differences from the corresponding active control groups; ## *p* < 0.0001—differences from the KCl group. *n* = 5–6 for each group. Preliminary administration of hLf increases the number of cells with induced c-Fos protein (**b**). The presence of hLf in the culture medium during and after stimulation does not significantly affect the number of c-Fos-positive cells. ## *p* < 0.0001—differences from the KCl group. *n* = 5–6 for each group (**c**). The data are presented as the mean ± SEM. PC—passive control, AC—active control.

In groups PC and PC+Lf, no c-Fos-positive cells were detected (Figure 6). The induction of this protein was found in ~15% of cells in both active control groups (AC, AC+Lf) 2 h after the medium change. Immunofluorescent analysis showed that cell stimulation with 50 mM KCl induced the c-Fos protein that significantly (*p* < 0.0001) differed from that in both passive and active controls (Figure 6b).

In addition, preliminary administration of hLf significantly increased the number of c-Fos-positive cells 2 h after application of 50 mM KCl (*p* < 0.0001): from 35% in group KCl to 50 and 47% in groups KCl+Lf and KCl+Lfconst, respectively (Figure 6b,c). The presence of hLf in the culture medium during and after stimulation did not significantly affect the number of c-Fos-positive cells (groups KCl+Lfconst and KCl+Lf) (Figure 6c).

## 3. Discussion

The biological effects of Lf are mediated by specific surface receptors on the target cells [28]. It was shown that the addition of exogenous Lf stimulated the expression of the Lf receptor in the primary culture of ventral midbrain neurons [29]. Lf rapidly entered the cytoplasm of cultured cells with the participation of receptor proteins LRP1 and nucleolin expressed in the cells [30,31,32,33]. Lf internalization into the nucleus was slower, and this process was mediated by nuclear nucleolin [32]. As nucleolin is a multiligand protein acting as a shuttle between the cell surface and the nucleus [30,34], many biological functions of Lf are presumably related to its binding with this receptor. In our study, similar effects of Lf (Figure 2a,b) were presumably mediated by the same receptors.

It has been demonstrated that the immunoreactivity of exogenous Lf in most cases is located in the cytoplasm [35]. After the addition of bovine Lf to the culture medium, the protein was detected in the cytoplasm of HT29 cells [36], HeLa cells [37], MCF-7 cells, primary bovine mammary epithelial cells [38], mouse L929 fibroblasts, and mouse GL-261 glioma cells [39]; nuclear localization of Lf was observed only in Caco-2 cells [36]. The synthesized pentapeptide corresponding to a fragment of the hLf N-terminal region enriched with basic amino acids a few minutes after addition to the culture medium was detected in the nucleus (mainly in the nucleoli) of HeLa cells, human glioblastoma U87MG cells, and human bladder carcinoma 5637 cells [40].

Lf can produce different effects depending on its cellular localization. For instance, in mouse GL-261 glioma cells, bovine Lf in the same dose exhibited a cytotoxic effect upon internalization into the cytoplasm and a proliferative effect after delivery to the nucleus with chitosan nanoparticles [39]. After the addition of bovine Lf to the culture medium, the protein was detected in the cytoplasm of MCF-7 cells and primary bovine mammary epithelial cells. However, in combination with retinoids, Lf can penetrate into the nucleus, which coincided with a decrease in their cytotoxic effects [38].

Stimulation, as a stress factor, can cause damage to neuronal cells in culture. The data obtained allow us to conclude that the applied stimulation scheme caused no additional damage to cells (Figure 3a,b); therefore, we studied the effect of hLf within the framework of a physiological response.

The expression of immediate early genes is initiated within a few minutes in response to the action of various stimuli on membrane receptors [41,42,43]. The dynamics of *c-fos* gene expression are known. The maximum level of c-Fos protein was observed 120 min after stimulation [23,44]. In hippocampal cell cultures, stimulation with 50 mM KCl induced the expression of c-Fos protein (observed after 2 h) that differed from the control levels (Figure 6). This is consistent with the data obtained by other authors. The expression of c-Fos protein was induced in pyramidal neurons of cultured rat hippocampal sections by triple application of 50 mM KCl [24] and in cultures of hippocampal neurons by the addition of 170 mM KCl [45].

Our data indicate that the preliminary administration of hLf enhanced the induction of transcription factor c-Fos in primary neuronal cultures in response to triple stimulation with 50 mM KCl, which in turn contributed to hLf translocation into the nuclei of cultured cells (Figure 6). Colocalization of hLf inclusion and c-Fos protein was observed in some nuclei (Figure 4b). There is convincing evidence that exogenous Lf can be translocated to the nucleus, where it can bind with DNA and act as a transcription activator. Lf has been shown to enhance the expression of brain-derived neurotrophic factor (BDNF) via the ERK/CREB pathway and the expression of polysialilated nerve cell adhesion molecule (PSA-NCAM) [10,11,46]. Lf added to the diet for 4 weeks increased the levels of mRNA for c-Fos, BDNF, and GluN1 subunit of the NMDA receptor in the prefrontal cortex of juvenile rats, but at the same time reduced the level of c-Fos mRNA in the amygdala [47]. Lf can trigger cell differentiation, activation, and proliferation [48]. It was shown that hLf promotes the differentiation of immature B and T cells and stimulates B cell maturation in mouse spleen [49,50], while bovine Lf modulates the differentiation and function of dendritic cells [51].

c-Fos protein is a genetic regulator of cellular mechanisms mediating the excitability and survival of neurons [19]. Our previous studies showed that hLf had a protective effect in the experimental model of neuronal death and promoted recovery of the functional activity of nigrostriatal system cells in mouse brain, which was manifested in an increase in the number of tyrosine hydroxylase-positive cells in the substantia nigra (from 36 to 53% relative to the control animals) and the optical density of tyrosine hydroxylase-positive fibers in the striatum (from 33 to 92% relative to the control animals) after acute exposure to the neurotoxin, MPTP [8,52]. In this study, the effect of hLf on the induction of transcription factor c-Fos in mouse hippocampal cell cultures was investigated for the first time and the colocalization of exogenous hLf with c-Fos protein after stimulation with 50 mM KCl was shown. Based on these reports and the results obtained in our study, we can assume that Lf modulation of the induction of the transcription factor c-Fos is a potential mechanism of long-term neuronal plasticity. For a more complete understanding of the involvement of Lf in the mechanisms of long-term plasticity, it is necessary to clarify the question of its possible role as a transcription factor.

## 4. Materials and Methods

Primary dissociated cultures of hippocampal cells were obtained from the brains of newborn C57BL/6 mice (P0-P1, *n* = 102) under sterile conditions. All manipulations were carried out in accordance with Directive 2010/63/EU on the Protection of Animals Used for Scientific Purposes [53] and requirements of the Local Ethical Committee on Biomedical Research of the Research Center “Kurchatov Institute” (Protocol No. 2, 6/26/2019). The hippocampi were extracted, crushed, and trypsinized (0.25%, 20 min, 37 °C; Sigma–Aldrich, St. Louis, MO, USA, T4049) [54,55]. The cells were washed twice in a complete culture medium, counted in a Goryaev chamber, and their concentration was adjusted to 3 × 10^6^/mL. Then, the cells (50 µL suspension) were seeded on glasses pretreated with 0.05% polyethylenimine (Supelco, Bellefonte, PA, USA) and placed in 35 mm Petri dishes (Greiner Bio-One, Frickenhausen, Germany). The seeding density was at least 1200 cells/mm^2^. The cell cultures were incubated at 37 °C, 100% humidity, and 5% CO_2_ (Galaxy 170 S incubator, New Brunswick Scientific, Edison, NJ, USA) in a Neurobasal medium (Gibco, 21103-049) containing B27 Supplement (2%, Gibco, 17504-044), L-glutamine (0.5 mM, Gibco, 25030-024), 50 U/mL penicillin, and 50 µg/mL streptomycin (Gibco, 15140-122, UK) [56]. The culture medium was half-replaced 24 h after seeding and then every 3 days.

Human Lf (Lactobio LLC, Moscow, Russia; purity 97%.) isolated from female colostrum was used in the study. The iron saturation of hLf was about 10%.

### 4.1. Experimental Groups and Stimulation

**Figure 7:**
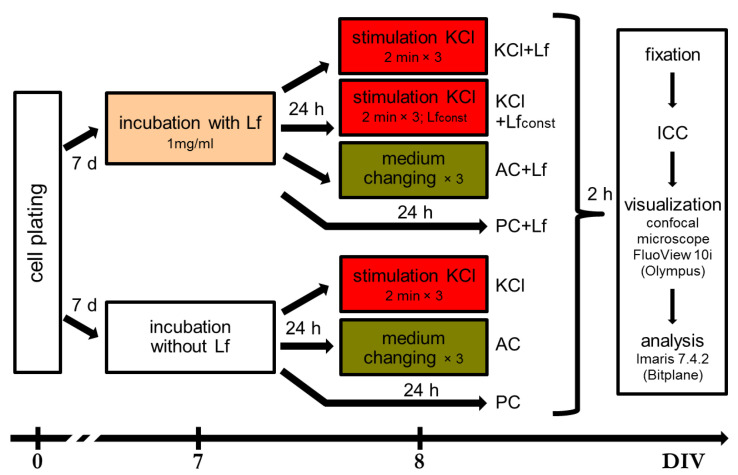
Experimental timeline. Primary dissociated cultures of hippocampal cells were obtained from the brains of newborn C57BL/6 mice (P0-P1). On day 7 of culturing, human Lf was added to the medium. After 24 h (day 8 in culture), c-Fos protein was induced in cells by triple application of 50 mM KCl. Cultures with 3-fold medium replacement were used as an active control (AC). The passive control group (PC) was fixed simultaneously with other cultures without additional manipulations. Immunocytochemical analysis (ICC) was performed 2 h after stimulation. DIV—day in vitro. PC—passive control, AC—active control.

Neuronal cultures were randomly divided into 7 groups: three experimental (KCl, KCl+Lf, and KCl+Lfconst, *n* = 10, 10, and 6, respectively), two active control groups (AC, AC+Lf, *n* = 6 in each), and two passive control groups (PC, PC+Lf, *n* = 9 and 12, respectively). On day 8 of culturing, c-Fos protein in cells was induced by adding 50 mM KCl (3 × 2 min with 5 min intervals) to the culture medium with complete medium replacement between the stimulations [24]. Cultures with 3-fold medium replacement were used as active controls. hLf was added to the culture medium of KCl+Lf, KCl+Lfconst, AC+Lf, and PC+Lf groups to a final concentration of 1 mg/mL 24 h before stimulation, medium changing, or fixation. The choice of this dose was based on published data [39] and the results of our pilot experiments. In the KCl+Lfconst group, the concentration of hLf during medium changes between the stimulations was maintained constant until the fixation of the cultures. The scheme of the experiment is shown in Figure 7.

Two hours after stimulation or medium change, the cultures were washed with 0.1 M PBS and fixed with 4% paraformaldehyde (Sigma–Aldrich, USA) in PBS (10 min on ice). This time point was chosen based on the work of Schoenenberger et al., where the same stimulation scheme was used [24]. The passive control groups were fixed simultaneously with other cultures without additional manipulations. Three independent experimental series were performed.

### 4.2. Evaluation of Genomic DNA Fragmentation in Cultured Neuronal Cells after Stimulation

DNA fragmentation was detected using a DNA Fragmentation In situ BrdU-Red (TUNEL) kit (Abcam, ab66110) according to the manufacturer’s instructions. The TUNEL method was based on fluorescent labeling of 3′-terminal hydroxyl groups in double-stranded DNA breaks with 5-Bromo-2′-deoxyuridine-5′-triphosphate (Br-dUTP) in a reaction catalyzed by exogenous terminal deoxynucleotidyl transferase (TdT), followed by detection using antibodies to bromodeoxyuridine labeled with a red fluorochrome [57,58].

Cultures of groups KCl (2 or 24 h after stimulation) and PC (*n* = 4 in each) were washed with 0.1 M PBS, fixed with 4% paraformaldehyde (Sigma–Aldrich, USA) in PBS (10 min on ice), then washed twice in PBS. The cells were incubated in PBS with 0.03% Triton X-100 for 10 min at room temperature for membrane permeabilization, washed in PBS for 10 min, and then, 100 µL washing buffer was applied (2 × 5 min). The cultures were incubated in the dark at 37 °C with TdT and Br-dUTP for 1 h, washed with PBS (2 × 5 min), stained with antibodies to bromodeoxyuridine labeled with red fluorochrome for 30 min at room temperature, and washed again with PBS for 5 min. Cell nuclei were poststained with DNA-specific dye Hoechst 33258 (1:1000; Invitrogen, H-1398). Stained cultures were digitized using a confocal microscope FV10i (Olympus, Tokyo, Japan) and analyzed using Imaris 7.4.2 (Bitplane, Switzerland) and Image-Pro Plus 6.0 software (Media Cybernetics, Rockville, MD, USA). The number of TUNEL-positive nuclei was expressed as a percentage of the total number of nuclei [27,59]; the arithmetic mean was calculated for 10–15 fields of view in each culture.

### 4.3. Dynamics of hLf Penetration into the Cytoplasm and Nucleus of Hippocampal Cells

**Figure 8:**
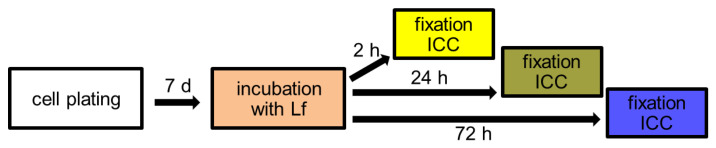
Experimental timeline for the analysis of the dynamics of human Lf penetration into the cytoplasm and nucleus of cells in the primary culture of the hippocampus. Primary dissociated cultures of hippocampal cells were obtained from the brains of newborn C57BL/6 mice (P0-P1). On day 8 of culturing, hLf was added to the medium. After 2, 24, and 72 h incubation, the cultures were washed and fixed, and immunocytochemical analysis (ICC) was performed using monoclonal antibodies to hLf.

On day 8 of culturing, hLf was added to the medium to a final concentration of 1 mg/mL. After 2-, 24-, or 72-h incubation (*n* = 6, 6, and 4, respectively) the cultures were washed with 0.1 M PBS and fixed with 4% paraformaldehyde (10 min on ice). The scheme of the experiment is shown in Figure 8. Immunofluorescent staining was performed using rabbit monoclonal antibodies (EPR4338) to Lf (1:150; Abcam, ab109000) followed by detection with donkey anti-rabbit antibodies labeled with Alexa fluorophore 568 (1:500; Abcam, ab175470). Cell nuclei were poststained with Hoechst 33258 (1:1000; Invitrogen, H-1398). Stained cultures were digitized and analyzed (see Section 4.2). The number of Lf-positive cells (with cytoplasmic or nuclear localization of the exogenous protein separately) was expressed as a percentage of the total number of nuclei; the arithmetic mean was calculated for 5–6 fields of view in each culture.

### 4.4. Immunocytochemical Detection of Proteins

Immunofluorescent staining was performed using goat polyclonal antibodies to c-Fos protein (1:200; Santa Cruz Biotechnology, sc-52-G), rabbit monoclonal antibodies (EPR4338) to hLf (1:150; Abcam, ab109000), and mouse monoclonal antibodies to NeuN (clone A60) (1:500; Millipore, MAB377) with subsequent detection with donkey anti-rabbit antibodies labeled with Alexa Fluor 488 fluorophore (1:500; Abcam, ab150129), donkey anti-rabbit antibodies labeled with Alexa Fluor 568 fluorophore (1:500; Abcam, ab175470), and donkey anti-mouse antibodies labeled with Alexa Fluor 647 fluorophore (1:500; Invitrogen, A-31571). Cell nuclei were poststained with Hoechst 33258 (1:1000; Invitrogen, H-1398). Stained cultures were digitized and analyzed (see Section 4.2). The number of c-Fos-positive nuclei was expressed as a percentage of the total number of nuclei [22]; the arithmetic mean was calculated for 10 fields of view in each culture.

### 4.5. Statistical Analysis

Statistical analysis was performed using GraphPad Prizm 8.0.1 software (La Jolla, San Diego, CA, USA). The normality of data distribution was assessed using the Shapiro–Wilk test. A one-way ANOVA was applied followed by the Tukey *post hoc* test or Šidák test for multiple comparisons. The data are presented as the mean ± SEM. *p* values < 0.05 were considered to be significant.

## 5. Conclusions

Stimulation promoted exogenous Lf translocation into the nuclei of cultured neuronal cells, which correlated with increased induction of transcription factor c-Fos and was accompanied by nuclear colocalization of these proteins. The results indicate the potential of Lf as a modulator of neuronal processes and open up new prospects in studying the mechanisms of the regulatory effects of lactoferrin on cell functions.

## Data Availability

Data are contained within the article.
